# The complete chloroplast genome sequence of *Platanthera chlorantha* (Orchidaceae)

**DOI:** 10.1080/23802359.2019.1644551

**Published:** 2019-07-22

**Authors:** Félix Lallemand, Michał May, Anna Ihnatowicz, Marcin Jąkalski

**Affiliations:** a Institut de Systématique, Evolution, Biodiversité (ISYEB), Muséum National d'Histoire Naturelle, CNRS, Sorbonne Université, EPHE, Paris, France;; b Department of Plant Taxonomy and Nature Conservation, Faculty of Biology, University of Gdańsk, Gdańsk, Poland;; c Intercollegiate Faculty of Biotechnology, University of Gdansk and Medical University of Gdansk, Gdańsk, Poland

**Keywords:** Chloroplast genome, *Platanthera chlorantha*, orchids, Orchidoideae

## Abstract

Here, we report the first complete chloroplast genome of *Platanthera chlorantha* (Orchidaceae: Orchidoideae). The circular genome with the length of 154,260 bp possesses the typical structure consisting of a large single copy region (LSC) of 83,279 bp and a small single copy region (SSC) of 17,759 bp, separated from each other by two copies of inverted repeats (IRs) of 26,611 bp. The plastome encodes 134 genes, of which 88 were protein-coding, eight encoded ribosomal RNA, and 38 transfer RNAs. The overall GC content was 36.74%. The plastome sequence provided here constitutes a valuable resource for analyzing genetic diversity of the *Orchidaceae* family.

Due to their extensive variability and biotic interactions, orchids present a compelling subject for evolutionary studies (Selosse 2014). Chloroplast genomes provide researchers with data invaluable for studying genetic history and phylogeny and thus were already used to resolve major phylogenetic relationships between orchid subfamilies (Givnish et al. 2015). In this study, we sequenced for the first time the plastome of *Platanthera chlorantha* (Custer) Rchb. This species inhabits mostly woods, meadows, and damp heaths and is widely spread through Europe, excluding eastern, northern, and south-western edges (Hultén and Fries 1986). It belongs to the *Orchidoideae* subfamily represented by almost 3800 species. With the main focus of researchers being put on the *Epidendroideae* tribes (142 of 175 orchid species with complete plastomes; NCBI GenBank, as of April 2019), currently only 14 complete chloroplast genomes are available within *Orchidoideae* tribes (Yu et al. 2015; Zhu et al. 2016; Roma et al. 2018; Oh et al. 2019) and only one of those belongs to the member of the same genus *Platanthera*, namely *P. japonica* (Dong et al. 2018).

Fresh plant leaves were collected from Kalina Lisiniec, Poland (50°21′43.7″N 20°09′37.4″E) and dried in silica-gel (voucher SG-13236, Herbarium of University of Gdansk, UGDA). Extraction of the total genomic DNA was performed using DNeasy Plant Mini Kit (Qiagen), followed by sequencing library generation with Accel-NGS^®^ 1S Plus DNA Library Kit (Swift Biosciences Inc., USA) and paired-end sequencing on Illumina HiSeq 4000. Geneious (version 10.2.4, https://www.geneious.com), with the Geneious assembling algorithm (medium-low sensitivity parameters) and a subset of 25% of the reads was used for plastome assembly. Annotations were obtained with Geneious annotations transfer procedure from *P. japonica* (NC_037440.1) with a 77% similarity threshold, additionally aided using GeSeq (Tillich et al. 2017) and followed by manual verification. Inferring of phylogenetic position of *P. chlorantha* was based on ML analysis performed with RAxML-NG (Kozlov et al. 2019) and multiple sequence alignment of full chloroplast sequences conducted with MAFFT (Katoh and Standley 2013).

The complete circular chloroplast genome of *P. chlorantha* (GenBank MK937914) is a 154,260 bp long sequence, with 36.74% GC content and a typical structure consisting of two inverted repeats (IRa and IRb) of 26,611 bp each, separated by a large single-copy region (LSC) of 83,279 bp and a small single-copy region (SSC) of 17,759 bp in length. The plastome contains 134 (113 unique) genes, of which eight are rRNA genes, 38 encode tRNAs, and 88 are protein-coding. One of the *ycf1* genes located at the IRb/SSC boundary is truncated but with complete open reading frame and one copy of the *rpl22* gene is a pseudogene. Introns were annotated for 12 protein-coding genes and six tRNA genes. Phylogenetic analyses (Figure 1) showed that *P. chlorantha* grouped together with *P. japonica* and other representatives of *Orchidoideae.* Despite floral similarities, it is phylogenetically distant from the genus *Habenaria* as already noted before (Bateman et al. 2003). In the genus *Platanthera*, some species display a trend to recover biomass from the symbiotic fungi colonizing their roots (i.e. a partial heterotrophy/mixotrophy, based on their mycorrhizal fungi; see Selosse and Roy 2009; Yagame et al. 2012; references therein). Our result show plastids with apparently intact photosynthetic abilities in *P. chlorantha*, which is in agreement with indirect evidence for autotrophy published by Bidartondo et al. (2004) for this species. The plastome sequence we provided here constitutes a valuable aid for analyzing the genetic diversity of the *Orchidaceae* family.

**Figure 1.**
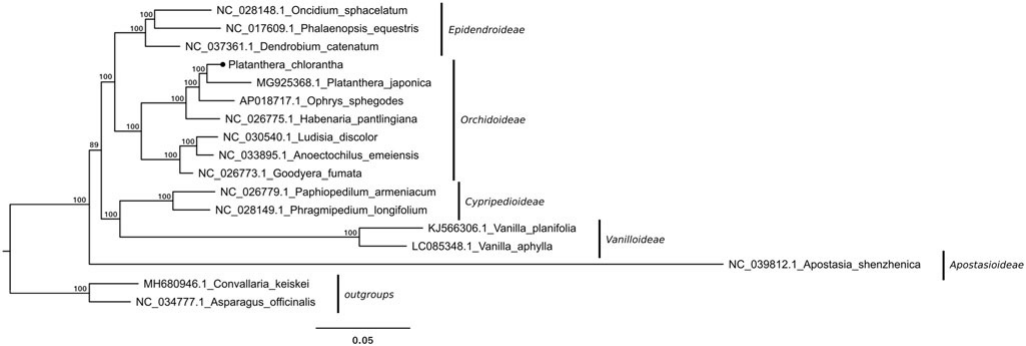
Maximum-likelihood-based phylogenetic tree of *Orchidaceae* representatives constructed using full length plastome sequences. The numbers on each node are RAxML bootstrap values (based on 1000 replicates). The tree is rooted with non-orchid monocot representatives.
